# Unveiling a Sour Truth: Acute Pancreatitis Linked to Apple Cider Vinegar Supplement

**DOI:** 10.14309/crj.0000000000001482

**Published:** 2024-09-05

**Authors:** Luis Alvarez, Lanson B. Colaco, Saba Ali, Iktej S. Singh Jabbal, Veerkaran Banga, Martin A. Avalos, Pankaj J. Patel

**Affiliations:** 1 AdventHealth Sebring, Sebring, FL

**Keywords:** apple cider vinegar supplements, pancreatitis

## Abstract

Acute pancreatitis, a common gastrointestinal ailment in the United States, often lacks a clear etiology, with one-third of cases deemed idiopathic. We discuss an 84-year-old woman with acute pancreatitis possibly linked to a recently introduced weight loss supplement containing apple cider vinegar. Literature review unveils scant data regarding the risks of acute pancreatitis associated with less rigorously studied and regulated supplements, such as apple cider vinegar products. Considering the morbidity and financial burden associated with acute pancreatitis, there is a pressing need to report and disseminate awareness of diverse etiologies, encompassing drug and supplement-induced cases. This case report endeavors to address this need.

## INTRODUCTION

Acute pancreatitis ranks among the primary reasons for hospital admissions due to gastrointestinal diseases, leading to around 300,000 visits to emergency departments annually.^1^ Almost one-third of cases of acute pancreatitis are labeled as “idiopathic,” with the other etiologies being alcohol consumption, gallstones, infections, autoimmune factors, and medications.^2,3^ We present a patient who developed acute pancreatitis after the consumption of a “weight-loss” supplement called “Ketobites Apple Cider Vinegar (ACV) gummies.” This is one of the few reported cases of a patient developing pancreatitis while on this supplement.

## CASE REPORT

An 84-year-old woman with a medical history of hypertension and osteoarthritis presented to the emergency department with an acute onset of epigastric pain, nausea, and vomiting for 2 days. The pain was described as a postprandial, sharp, 7/10 in intensity, localized to the epigastric area, and notably exacerbated by palpation. This was associated with nausea and vomiting. She denied any prior episodes of a similar nature, history of gallstones, heavy alcohol consumption, unintentional weight loss, hypertriglyceridemia, or trauma to the abdomen. Her home medications included ibuprofen, metoprolol-succinate, losartan-hydrochlorothiazide, and alprazolam, all of which she had been taking for over 1 year. The patient's body mass index was recorded at 27.

**Figure 1.**
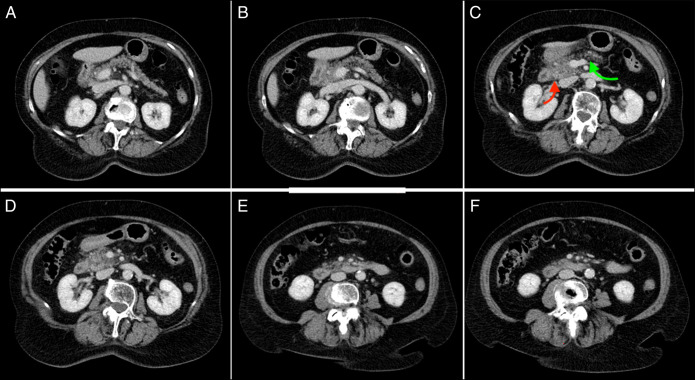
(A–F) Computed tomography abdomen findings. Red arrow denotes inflamed pancreas, and green arrow denotes normal pancreas.

Upon examination, her vitals were stable except for hypertension (149/89 mm of mercury) and tachycardia (104 beats per minute). Laboratory analysis revealed leukocytosis (18,700 cells/µL), thereby meeting the criteria for systemic inflammatory response syndrome. The lipase level was mildly elevated at 113 units/L (reference range 8–57), and abdominal ultrasound did not show evidence of cholelithiasis. Triage laboratory findings have been highlighted in Table 1. A computed tomography scan of the abdomen and pelvis (Figure 1) identified subtle peripancreatic edema centered around the pancreatic head. These findings, along with the profound epigastric pain and tenderness, led to a diagnosis of acute pancreatitis. The patient's Ranson score at admission was 2 points, suggesting a 1% mortality risk and making severe pancreatitis unlikely.

**Table 1.** Triage laboratory findings on presentation

Laboratory test	Value	Units	Reference range
Hemoglobin	14.3	g/d	13.5–17.5 (male), 12.0–15.5 (female)
Hematocrit	39.9	%	38.3–48.6 (male), 35.5–44.9 (female)
Red blood cells	4.64	Million/µL	4.7–6.1 (male), 4.2–5.4 (female)
White blood cells	16.6	Thousand/µL	4.0–11.0
Platelets	274	Thousand/µL	150–450
Sodium (Na)	132	mEq/L	135–145
Potassium	3.7	mEq/L	3.5–5.0
Bicarbonate	22.5	mEq/L	22–29
Chloride	95	mEq/L	96–106
Glucose	139	mg/dL	70–100 (fasting)
Blood urea nitrate	9.9	mg/dL	7–20
Creatinine	0.48	mg/dL	0.6–1.2 (male), 0.5–1.1 (female)
Aspartate aminotransferase	21	U/L	10–40
Alkaline aminotransferase	12	U/L	7–56
Alkaline phosphatase	92	U/L	44–147
Total protein	7.6	g/dL	6.0–8.3
Albumin	4.3	g/dL	3.5–5.0
Globulin	3.3	g/dL	2.0–3.5
Total bilirubin	1.00	mg/dL	0.1–1.2
Troponin	<12	ng/L	<14
Lactic acid	1.11	mmol/L	0.5–1.9
Lipase (daily trend)	113, 600, 600, 111, 65, 81	U/L	0—57
Procalcitonin	0.04	ng/mL	<0.1
Triglycerides	43	mg/dL	<150
Calcium	9.8	mg/dL	8.4–10.2

**Figure 2.**
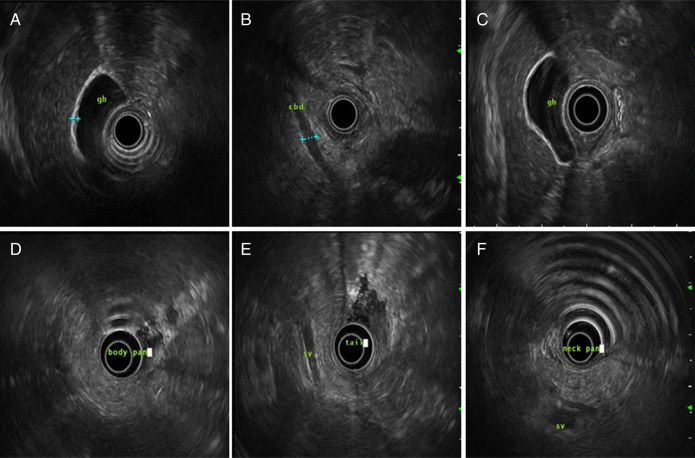
(A–C) EGD and EUS revealed a nondilated common bile duct, normal gallbladder, without stones/sludge. (D–F) EGD and EUS show normal pancreas. EGD, esophagogastroduodenoscopy; EUS, endoscopic ultrasound.

The patient was provided symptomatic management, including supportive care with fluid resuscitation and pain control. Early feeding was initiated 24 hours postadmission. The initial evaluation of the etiology ruled out alcohol use, hypertriglyceridemia, hypercalcemia, and gallstones as potential causes of the patient's acute pancreatitis. This led to the consideration of medication-induced causes. A thorough reconciliation of the patient's medications revealed recent daily consumption of a weight-loss supplement known as “Ketobites ACV gummies” for the past 30 days. Esophagogastroduodenoscopy with endoscopic ultrasound (Figure 2) revealed a normal common bile duct, pancreatic duct, and pancreatic parenchyma. The patient was advised to avoid consuming similar medications in the future. The thiazide medication was also discontinued, and the patient was discharged with recommendations for outpatient follow-up.

## DISCUSSION

Acute pancreatitis imposes a substantial financial burden on the U.S. healthcare system, amounting to $9.3 billion annually and exhibiting a persistent upward trend.^4^ Most cases can be attributed to alcohol consumption, gallstones, infections, autoimmune factors, and medications, while almost one-third of cases are categorized as idiopathic.^3^ The absence of a clear etiology heightens the likelihood of recurrence due to the absence of a known trigger.

There is a clear need for preventative measures against the development of recurrent pancreatitis, and thus, identifying the etiology of acute pancreatitis must be a paramount focus. Drug-induced pancreatitis alone accounts for 5% of all acute pancreatitis cases. The World Health Organization has documented over 500 drugs that induce acute pancreatitis. Several patients may have multiple contributing etiologies; therefore, definitively attributing acute pancreatitis to a specific drug poses considerable challenges. Consequently, physicians may easily overlook the potential for new drugs or supplements to induce acute pancreatitis, leading to the classification of cases as idiopathic. In our case, the patient was on losartan-hydrochlorothiazide for more than a year, a recognized but rare cause of pancreatitis. The induction mechanism typically involves hypercalcemia or hypertriglyceridemia, which the patient in question did not exhibit.^5^ Moreover, the patient had been tolerating the drug well for well over a year before the development of pancreatitis. This was, therefore, ruled out as a cause.

The only recent change reported by the patient was the use of a “keto” supplement for 30 days (while not practicing a keto diet), promoted on social media for weight loss, with its primary ingredient being 1,000 mg of ACV. Social media and business entities are marketing vinegar products as natural appetite suppressants, fostering the perception among some individuals that vinegar serves as a healthy daily supplement for weight loss. Literature supports the idea that vinegar significantly increases satiety.^6^ However, it is crucial to note that despite these purported benefits, several cases in the literature highlight potential risks associated with vinegar ingestion. These include esophageal injury, biochemical disturbances, dental injury, and, more rarely, acute pancreatitis.^6^ For instance, there is a documented case where a patient developed acute pancreatitis after ingesting 2 tablespoons of acetic vinegar daily for 2 weeks.^7^ Notably, our case involves an ACV supplement, and, to our knowledge, there are no reported instances in the literature of such a supplement inducing pancreatitis.

Distinguishing between drug-induced pancreatitis and idiopathic pancreatitis presents a formidable clinical hurdle. In this instance, a previously healthy patient developed acute pancreatitis with no apparent cause other than the recent addition of an ACV supplement. The emergence of supplement-related complications highlights the importance of considering unconventional etiologies, particularly in light of the pervasive advertising promoting non-Food and Drug Administration–approved supplements. Case reports like this one underscore the need for heightened awareness among healthcare providers regarding the potential adverse effects of supplement use. As evidence accumulates, advocating for an evidence-based approach to supplement sales and consumption becomes increasingly imperative, promising improved patient outcomes.

## DISCLOSURES

Author contributions: L. Alvarez, LB Colaco, S. Ali, ISS Jabbal, PJ Patel, V. Banga: Substantial contributions to the conception or design of the work or the acquisition, analysis, or interpretation of data for the work. L. Alvarez, LB Colaco, S. Ali, ISS Jabbal, V. Banga: Drafting the work or reviewing it critically for important intellectual content. MA Avalos, PJ Patel, LB Colaco, ISS Jabbal, V. Banga: Final approval of the version to be published. L. Alvarez, LB Colaco, S. Ali, ISS Jabbal, PJ Patel, MA Avalos, V. Banga: Agreement to be accountable for all aspects of the work in ensuring that questions related to the accuracy or integrity of any part of the work are appropriately investigated and resolved. PJ Patel is the article guarantor.

Financial disclosure: None to report.

Previous presentation: This report has been accepted for poster presentation at the Florida Medical Association, David A. Paulus, MD Poster Symposium held during their Annual Meeting, August 2–4, 2024, Tallahassee, FL.

Informed consent was obtained for this case report.
